# Toxicity Assessment of Brazilian Brown Propolis-Cu^2+^ Complex with Enhanced Visible-Light Absorption and Antimicrobial Properties

**DOI:** 10.1021/acsomega.5c07268

**Published:** 2025-11-13

**Authors:** Douglas A. Guibes, Jhonatan M. P. Rocha, Patrícia Appelt, Mário A. A. da Cunha, Rogério P. Mateus, Luciana P. B. Machado, Daiane F. Ferreira

**Affiliations:** † Chemistry Department, 307046 Universidade Estadual do Centro-Oeste Campus CEDETEG, Elio Antônio Dalla Vecchia Lane, 838, Vila Carli, CEP 85040-167 Guarapuava, Paraná, Brazil; ‡ Chemistry Department, Universidade Tecnológica Federal do Paraná (UTFPR) Campus Pato Branco, Via do Conhecimento, s/n, Fraron, CEP 85503-390 Pato Branco, Paraná, Brazil; § Biological Sciences Department, Universidade Estadual do Centro-Oeste Campus CEDETEG, Elio Antônio Dalla Vec-chia Lane, 838, Vila Carli, CEP 85040-167 Guarapuava, Paraná, Brazil

## Abstract

In this study, we synthesized and characterized a novel Propolis-Cu (II) complex to enhance its photophysical and antimicrobial properties. The complex was characterized using UV–vis spectroscopy, FTIR, and scanning electron microscopy (SEM), revealing a distinct absorption profile and significantly enhancing Cu^2+^ absorption bands at 800–900 nm. FTIR reveals molecular interactions via carbonyl inhibition, and SEM indicate morphological changes with distinct crystalline structure formation. Although the Cu-PRO exhibits a significant decrease in antioxidant activity compared to propolis extract, the complex still retains considerable activity, which could be beneficial in future therapeutic applications. The antimicrobial activity of the Cu-PRO was significantly enhanced compared to pure propolis. While propolis alone exhibited strong activity against *Escherichia coli* (MIC = 12.5 μg·mL^–1^) and good activity against *Listeria monocytogenes* (50.0 μg·mL^–1^), the complex demonstrated an enhanced bactericidal action against *Staphylococcus aureus* at lower concentrations (50.0 μg·mL^–1^) and good inhibitory activity against *Salmonellagallinarum* (50.0 μg·mL^–1^) but not in *E. coli* and *L. monocytogenes*. Critically, toxicological assessment in *Drosophila melanogaster* revealed a chronic and dose-dependent toxicity profile, with lethal effects emerging only at doses >2.5 mg·mL^–1^, in longer exposure, and a cumulative reduction in LC_50_ over time (7.92 → 3.64 mg·mL^–1^ in 6 days). However, toxicity was expressively lower than free Cu^2+^ salts, attributed to propolis-mediated chelation reducing free Cu^2+^ bioavailability and antioxidant activity, possibly mitigating oxidative stress. The complex’s improved antimicrobial properties against *S. aureus* and *S. gallinarum*, coupled with modulated toxicity, position it as a promising candidate for therapeutic applications; particularly, its enhanced visible-light absorption in 800–900 nm falls into the therapeutic window of Photodynamic Therapy (PDT). We speculate that the results presented here highlight the potential of this complex as an innovative approach for combined therapies. Future studies are focusing on exploring its mechanisms of action and potential for light-assisted therapies, such as PDT where it can be used to simultaneously promote wound healing and combat infections.

## Graphical abstract

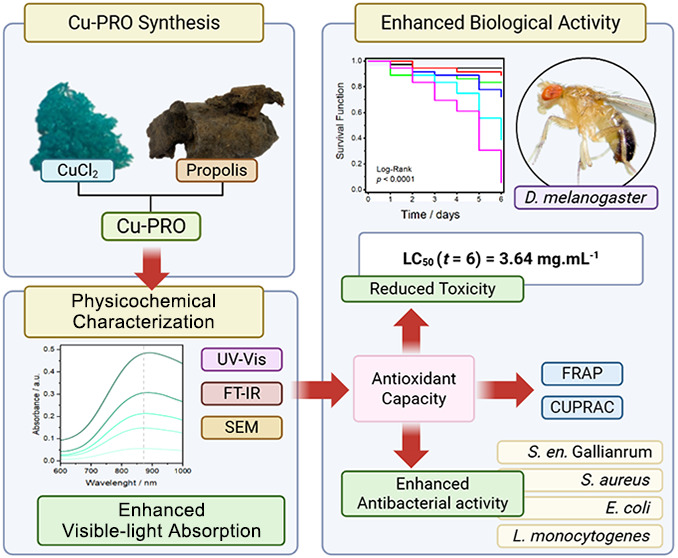

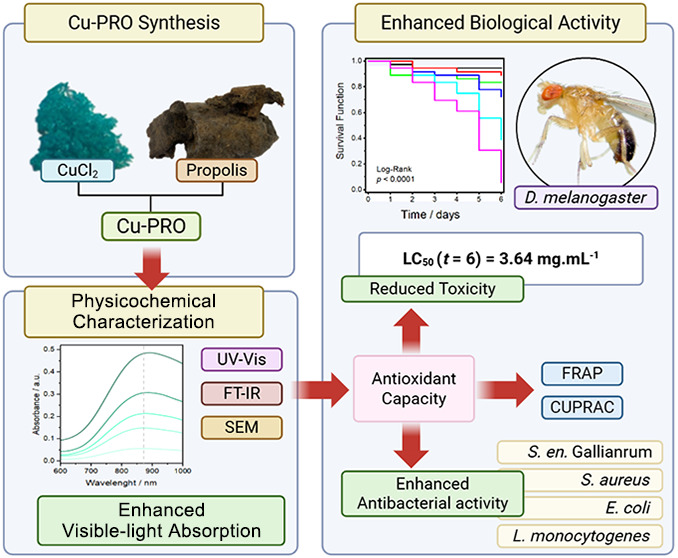

## 1 Introduction

Originating from the Greek language, the word “*propolis*” is composed of “*pro-*” meaning “in favor of,” and “*polis*,” referring to “city,” symbolizing the protection of the hive. The propolis produced by bees (Figure 1) serves structural and defensive purposes for the hive.^1^ It maintains the hive’s internal temperature, seals cracks, prevents predator invasions, and ensures an aseptic environment.^2,3^ Propolis has been used for medicinal purposes since ancient civilizations, with its benefits recognized as early as 300 B.C.^2,4^

**Figure 1:**
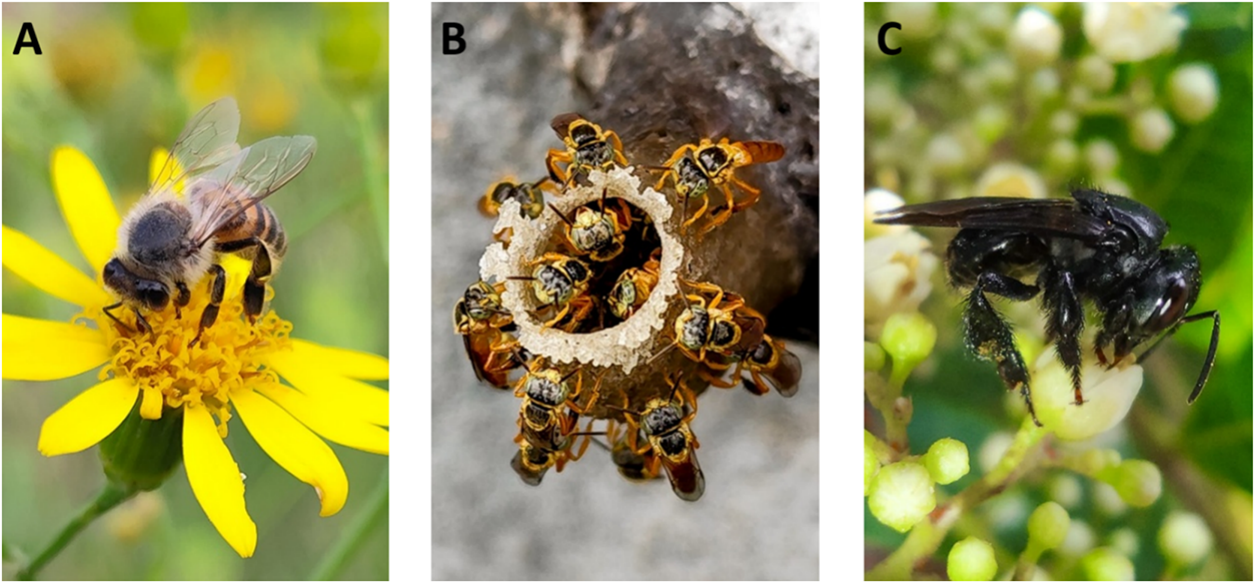
Some bees from the Apidae family known for propolis production: (A) *Apis mellifera* (Apini), (B) *Tetragonisca fiebrigi* (Meliponini), and (C) *Scaptotrigona bipunctata* (Meliponini) (Photographs courtesy of the author, Jhonatan M. P. Rocha, 2024).

Propolis is a resinous substance prepared by bees from a mixture of plant exudates, salivary secretions, and waxes.^3,5^ In its raw form, propolis consists of approximately 50% resins and aromatic balsams, 25–35% waxes, 10% essential oils, and 5% pollen.^1–3^ Its chemical composition varies, depending on the local flora. Over 800 different compounds have been identified, including aldehydes, ketones, phenolic acids, minerals, esters, flavonoids, vitamins, and alcohols.^6–8^ The pharmacological properties of propolis, such as antitumor, antibacterial, antifungal, antioxidant, and anesthetic effects, are attributed to its composition.^1,9–12^

Among the benefits of propolis, its ability to accelerate wound healing has stood out. Its antiallergic, anti-inflammatory, and antimicrobial properties, combined with phenolic compounds and enzymes that promote collagen formation and reduce free radical production, facilitate tissue regeneration.^3,10,13,14^ Propolis has shown promising results in treating chronic wounds, such as ulcers, providing patients with improved quality of life through accessible and effective methods.^15^ In this context, propolis is a promising material for application or association with new alternative methods in treating such injuries.^1,9–12^

Propolis has been associated with novel and alternative therapeutical methods that can take advantage of its extensive medicinal and biological proprieties. One of those is photodynamic therapy (PDT), a treatment that destroys targeted tissues through photochemical reactions. This technique involves three fundamental elements: a photosensitizer, a light source, and molecular oxygen. The activation of the photosensitizer by light in the presence of O_2_ generates reactive oxygen species (ROS), which are highly toxic to cells, resulting in therapeutic effects.^16,17^ PDT has gained prominence in treating chronic wounds, particularly in combating bacterial biofilms, which is a common barrier in chronic wounds. These biofilms hinder the action of systemically administered antibiotics.^18^

A recent study by Ribeiro et al.^19^ applied Brazilian Green Propolis and blue LED in antimicrobial PDT to treat intradermal infection of methicillin-resistant *Staphylococcus aureus* in a murine model, demonstrating effective preliminary results by significantly reducing the bacterial load at the infection site. Despite this study, most variants of propolis only has shown positive synergistic effects when combined with PDT, but its application directly as a photosensitizer tends to be limited due to low absorbance in the visible region, which is essential for an effective photosensitizer.^20^

Most transition metal compounds, such as copper, exhibit absorption in the visible region. Copper nanoparticles, for example, have been applied synergically in PDT to cancer treatment.^21^ The complexation of Cu^2+^ with propolis emerges as a strategy not only to amplify this visible-light absorption but also to modulate the metal’s toxicity and enhance its biological properties. This synergy can be particularly relevant for further applications such as an adjunct to PDT, where visible-light absorption is crucial to controllably generate cytotoxic ROS.

However, the Cu^2+^ ion is associated with toxicity mechanisms mediated primarily by excessive ROS generation,^22–24^ which triggers oxidative stress, damage to biomolecules such as DNA, proteins, and lipids.^22,24^ Recently, a programmed cell death mechanism dependent on intracellular Cu^2+^ accumulation, cuproptosis, was discovered.^23,24^ The Cu^2+^ reduction to Cu^+^ by ferredoxin-1 leads to aggregation of lipoylated proteins in the Krebs cycle, resulting in mitochondrial collapse.^23^ Although toxic in uncontrolled contexts, this mechanism has been explored as a therapeutic opportunity for the selective induction of cuproptosis in tumor cells.^24^

In biological models such as *Drosophila melanogaster*, widely used in toxicity studies due to their genetic similarity to humans, Abolaji et al.^25^ demonstrated that chelation of Cu^2+^ by D-penicillamine significantly reduces its toxicity, prolonging survival in exposure scenarios, related to the ability of ligands to sequester free ions, mitigate oxidative stress, and facilitate excretion. Similarly, Brazilian propolis, rich in phenolic compounds and flavonoids^4,8,26^ with chelating sites,^27^ can act as a natural agent to form stable complexes with Cu^2+^, reducing its bioavailability and toxicity, suppress uncontrolled ROS generation through its high antioxidant activity,^28^ and preserve its biological activities while enhancing its visible absorption.

In this context, this manuscript reports on the reaction between the extract of Brazilian brown propolis and Cu^2+^, targeting the synthesis of a novel complex that combine the bioactive properties of propolis with the electronic features of copper, alongside a toxicological assessment in *D. melanogaster* model was used to ensure preliminary toxicological safety. Bridging material design and biological validation, offering a sustainable strategy for developing natural hybrid compounds for advanced therapeutics.

## 2 Methodology

### 2.1 Propolis Complexation

The raw brown propolis (*A. mellifera*) was provided by Entreposto Colmeia Real Ltd., Prudentópolis-PR, Brazil, in 2021. The ultrapure water used was obtained using a Merck Millipore Milli-Q filtration system.

The propolis extract was prepared via ultrasound-assisted maceration, adapted from Contieri et al.^29^ A 100 g portion of raw brown propolis was mixed with 300 mL of a 70% (v/v) hydroalcoholic solution (95% ethanol, NEON) and subjected to ultrasonication (750 W) for 30 min. After sonication, the mixture was left to rest for 48 h, followed by an additional 30 min of ultrasonication. The mixture was then vacuum-filtered. The filtrate was stored in a freezer for 24 h and vacuum-filtered again to remove waxes. The crude extract was dried by using a rotary evaporator. The resulting solid was resuspended in a 70% (v/v) hydroalcoholic solution at a concentration of 20 mg·mL^–1^.

A 5% (m/v) solution of CuCl_2_·2H_2_0 (Sigma-Aldrich) was prepared by using ultrapure water. The complexation reaction was carried out in a 100 mL volumetric flask, where 10 mL of CuCl_2_ solution and 5 mL of propolis extract were added, and the flask was filled to volume with 95% (v/v) ethanol. The product was dried in an oven at 40 °C for 24 h.

### 2.2 Physicochemical Characterization

The UV–vis absorption spectra were obtained using a Shimadzu UV-1800 spectrophotometer, ranging from 200 to 900 nm, using quartz cuvettes. The samples were analyzed in a solution. The propolis extract and the CuCl_2_ solution were diluted by 100 μL to a 25 mL volumetric flask. An aliquot of 50 μL was added to a 10 mL volumetric flask for the Cu-PRO complex. The volumes were adjusted with ultrapure water for CuCl_2_ and 95% ethanol for the other samples. The extinction coefficient (ε), in mL·mg^–1^·cm^–1^, of the complex band was determined by linear regression varying the complex concentration in a range of 0.2–1.5 mg·mL^–1^, using the Lambert–Beer law (eq 1), where *A* is the absorbance in a concentration *C* and *b* is the optical cell length (1.0 cm).

1 A=ε·b·C

Vibrational infrared (FTIR) spectra were obtained using a PerkinElmer Frontier spectrophotometer (PerkinElmer, Waltham, MA) in Attenuated Total Reflectance (ATR) mode, in the range of 4000–650 cm^–1^, using solid samples. Morphological analysis was performed using a Hitachi TM-3000 scanning electron microscope (Hitachi, Irving, TX), also with solid samples.

### 2.3 *In Vitro* Antioxidant Activity

#### 2.3.1 Cupric Reducing Antioxidant Capacity (CUPRAC)

The methodology was adapted from Apak et al.^30^ In a test tube, 50 μL of the sample was added, followed by 1000 μL of an aqueous solution of CuCl_2_·2H_2_O (0.01 mol·L^–1^), 1000 μL of an ethanolic solution of neocuproine (0.0075 mol·L^–1^, Sigma-Aldrich), 1000 μL of an aqueous solution of ammonium acetate (1.0 mol·L^–1^, pH ∼ 7.02, Sigma-Aldrich), and 1050 μL of ultrapure water, totaling 4050 μL. The reaction was conducted in the dark for 30 min, followed by absorbance measurement at 450 nm. Ascorbic acid (Sigma-Aldrich) was used as the standard antioxidant substance, and a 70% (v/v) aqueous ethanol solution was used as the control. The entire procedure was performed in triplicate and protected from light.

2 $$total\;equivalence=\frac{E}{C\cdot V}$$

The total equivalence was calculated using eq 2, where *E* is the concentration equivalence calculated from the calibration curve (μg·mL^–1^), and *C*·*V* represents the mass content of the sample in the reaction, given by the product of the initial sample concentration (*C*) and the volume of the sample used (*V*). The final content was expressed in milligrams of antioxidant substances equivalent to ascorbic acid per gram of sample (mg of AAE·g^–1^).

#### 2.3.2 Ferric Reducing Antioxidant Power (FRAP)

The methodology was adapted from Rufino et al.^31^ For the preparation of 250 mL of the FRAP reagent, the following were mixed at the time of analysis: 208.30 mL of 0.3 M acetate buffer (pH ∼ 3.6), 20.80 mL of an aqueous solution of 2,4,6-Tris­(2-pyridyl)-*s*-triazine (TPTZ) (10 mmol L^–1^, Sigma-Aldrich), and 20.80 mL of an aqueous solution of FeCl_3_·6H_2_O (20 mmol L^–1^, Sigma-Aldrich).

In test tubes, 90 μL of the sample and 270 μL of ultrapure water were added, followed by 2700 μL of the FRAP solution. The mixture was homogenized using a vortex and kept in a thermostatic bath at 37 °C for 30 min, protected from light. After this period, the absorbance was measured at 595 nm.

A solution of Fe^2+^ (FeSO_4_·7H_2_O, 2.0 mM) was used as a standard for ferric ion-reducing substances. Ultrapure water was used as the blank. The ferric ion-reducing activity was calculated using eq 1, expressed in millimolar equivalents of Fe^2+^ per gram of sample (mMFeE·g^–1^).

### 2.4 Antimicrobial Activity

The minimum inhibitory concentration (MIC) method was performed using serial microdilution in a 96-well plate, following the Clinical and Laboratory Standards Institute (CLSI) guidelines.^32,33^ The samples, propolis extract, CuCl_2_, and Cu-PRO, were tested against bacterial strains, including Gram-positive bacteria: *S. aureus* (ATCC 25923) and *Listeria monocytogenes* (ATCC 19111); and Gram-negative bacteria: *Escherichia coli* (ATCC 25922) and *Salmonella enterica* serovar Gallinarum (ATCC 9184). The inoculum concentration was adjusted using the McFarland scale and measured with a spectrophotometer (PerkinElmer, Waltham, MA) at 625 nm to achieve a final concentration of 1.5 ×10^8^ CFU·mL^–1^.

The samples were prepared at 200 μg·mL^–1^ (w/v) concentration in 10% ethanol. After serial dilution, 100 μL of culture medium (Mueller-Hinton) and 20 μL of inoculum were added into the 96 wells of the ELISA microplates, followed by incubation at 37 °C for 24 h. After incubation, 20 μL of 2,3,5-triphenyltetrazolium chloride (0.125% w/v in ethanol) was added to each well, and the plates were incubated for an additional 2 h, after which the MIC was determined. Chloramphenicol (1.2 mg·mL^–1^) served as a positive control, 10% (v/v) ethanol as the negative control, and the culture medium alone as the growth control.

In wells ≥MIC, the minimum bactericide concentration (MBC) was assessed to determine the minimum concentration at which colony growth is present or absent in the culture medium. The minimum concentration that inhibited colony growth was classified as bactericidal, while those that still allowed growth were classified as bacteriostatic.

### 2.5 *In Vivo* Toxicity in the *D. melanogaster* Model

This test was performed by using the Canton-S (CS) wild-type strain of *D. melanogaster*. The individuals were treated in a transparent glass vial containing culture medium, banana-agar, and capped with cotton. The vials were kept at 25 °C under natural daylight cycle. The full procedure was adapted from Kumari et al.^34^

A doped culture medium was prepared by standardizing its volume (10 mL) and mixing it with a Cu-PRO solution in 500 μL of 50% ethanol. The concentration range studied was 0 (control), 0.1, 1.3, 2.5, 3.8, and 5.0 mg·mL^–1^, in triplicate (12 flies in each replica). As a control, the culture medium with 500 μL of 50% ethanol was used. Before introducing the individuals into the vials containing the extract, the flies were kept fasting in an empty vial for 3 h. Female flies (of varying ages) were used per replicate, totaling 216 individuals. Mortality was observed daily in specific hours for a period of 6 days, in time-dose variation.

The cumulative mortality data were processed into binary data (0 censoring, 1 event). Survival analysis was conducted using Kaplan–Meier estimates and tested with a log-rank (Mentel-Cox) test, general and pairwise, to compare survival across the doses. Parametric survival models (Exponential, Weibull, Log-normal, Log–logistic, Standard γ and Generalized γ) were fitted, and the model with the lowest Akaike Information Criteria (AIC) and the most adequate Cox-Snell residual was selected to estimate its parameters and the LC_50_ (lethal concentration for 50% of the population) during experimental period. All analyses were performed in R (v. 4.4.2).

## 3 Results and Discussion

As observed in Figure 2A,B, the initial colors of the CuCl_2_ solution and the propolis extract are blue and brown, respectively. A color change occurred in the resulting solution, which turned green after the reaction (Figure 2C), distinguishing it from its precursor materials.

**Figure 2:**
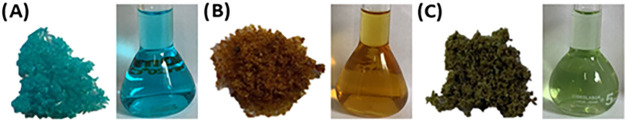
Coloring of solid and solution samples: (A) CuCl_2_, (B) brown propolis extract, and (C) Cu-PRO complex (Photographs courtesy of the author, Douglas A. Guibes, 2024).

### 3.1 Physicochemical Characterization

#### 3.1.1 UV–Vis Spectroscopy

According to Paganotti et al.^35^ and Tomazzoli et al.,^36^ since propolis is rich in phenolic compounds, particularly flavonoids, it tends to exhibit characteristic absorption bands closer to the ultraviolet region. As observed in Figure 3A, propolis shows two absorption bands at 230 nm and another band between 270 and 330 nm. This is a typical profile for propolis samples, as previously reported by Barbeira et al.^37^ Regarding the spectrum of pure CuCl_2_, intense absorption is observed below 230 nm.

**Figure 3:**
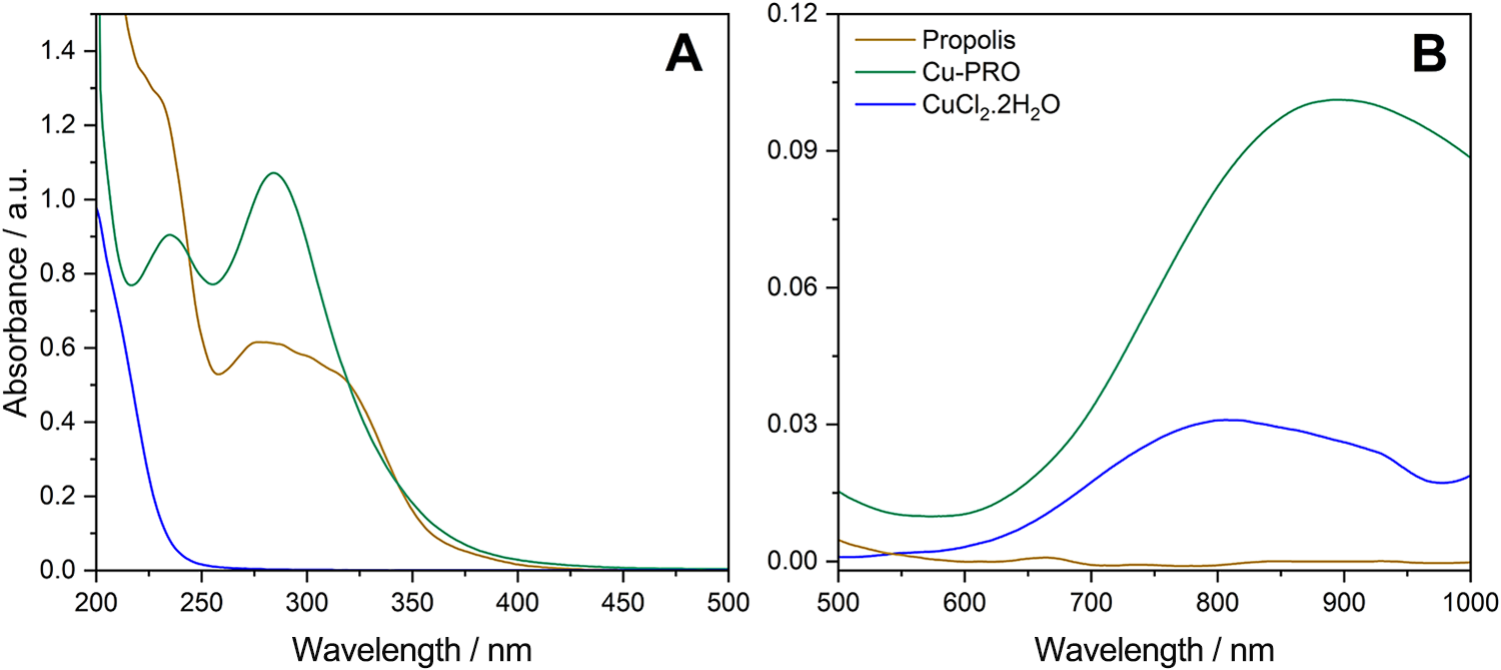
UV–vis absorption spectra of the samples in the reading ranges: (A) 200–400 nm (in 0.1 mg·mL^–1^) and (B) 400–1000 nm (0.35 mg·mL^–1^) (Panels (A, B) are shown on different scales due to very intense characteristic absorption in UV, even at very low concentrations).

The Cu-PRO complex caused a shift in the absorption profile of propolis, with both bands moving to lower wavelengths (λ). The observed changes suggest the occurrence of complexation between propolis and Cu^2+^, as this region is characteristic of propolis. The spectra obtained in the 400–900 nm region (Figure 3B) show that propolis has only one low-intensity absorption band at 660 nm, which is suppressed after complexation.

The reaction product exhibits enhanced absorption bands at 800 and 870 nm, when compared to pure CuCl_2_, though slightly shifted, which has its maximum at 800 nm, and the Cu-PRO around 872 nm. The formation of this intensified band of absorption is a significant indication for potential photophysical applications such as PDT, as its λ_max_ falls within its therapeutic window. Despite that, the extinction coefficient (ε), as shown in Figure 4, of the Cu-PRO band (872 nm) exhibits a mild value 0.34 mL·mg^–1^·cm^–1^ (*R* ^2^ = 98.8%) that can be effective in topical use. However, this serves only as speculation, as further studies are in development to evaluate and validate its activity as a topical photosensitizer.

**Figure 4:**
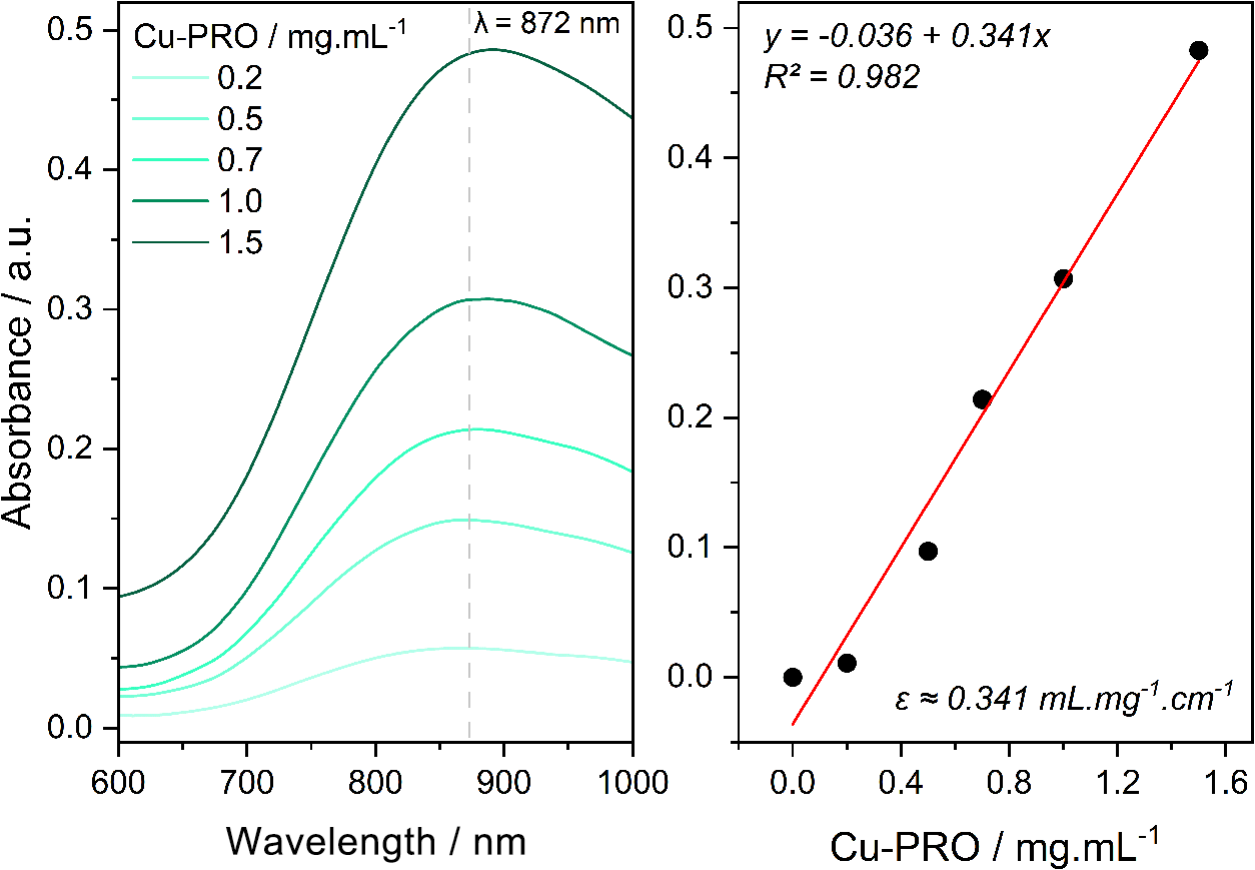
Determination of extinction coefficient in the Cu-PRO visible band.

In summary, the spectral shifts observed in the UV–vis profile of the Cu-PRO complex, particularly the intensification of Cu^2+^ absorption bands while coordinated, confirm successful complexation between propolis and Cu^2+^. While these findings highlight promising photophysical properties, further studies are essential to empirically validate its photosensitizing efficacy, stability under irradiation, and biological safety in realistic therapeutic scenarios.

#### 3.1.2 Infrared Spectroscopy (FTIR)

FTIR analysis was performed to investigate the samples’ molecular interactions and compare the precursors with the Cu-PRO complex. The observed vibrations in the spectra (Figure 5) are as follows: The propolis spectrum exhibits characteristic bands for hydroxyl groups (O–H; 3300 cm^–1^), methyl groups (C–H_3_; 2800 and 2850 cm^–1^), and carbonyl groups (C=O; 1600 cm^–1^). In the CuCl_2_ spectrum, bands associated with O–H stretching (3300 and 3150 cm^–1^) from water molecules and possibly Cu–O bending from coordinated water are observed.^38,39^

**Figure 5:**
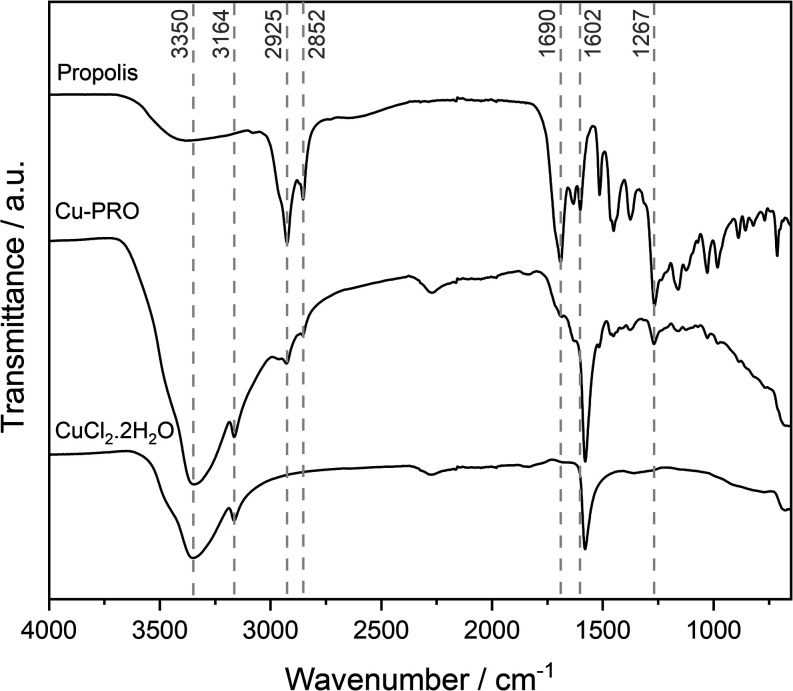
Vibrational infrared (ATR) spectra of Cu-PRO and precursor materials.

Differences in band shifts and intensities are observed in the Cu-PRO product compared to those of the starting materials. The signal at 1600 cm^–1^, corresponding to the carbonyl group, is inhibited in the Cu-PRO complex, which may indicate a possible site of complexation. At 1579 cm^–1^, there is a slight band shift compared to CuCl_2_. The bands at 1377 cm^–1^, characteristic of the C–O bond, and at 1270 cm^–1^, characteristic of ether groups (C–O–C), show minor variations compared to those of propolis. We can suggest the following possible structures for the complexation of propolis flavonoids with Cu^2+^, as shown in Figure 6.

**Figure 6:**
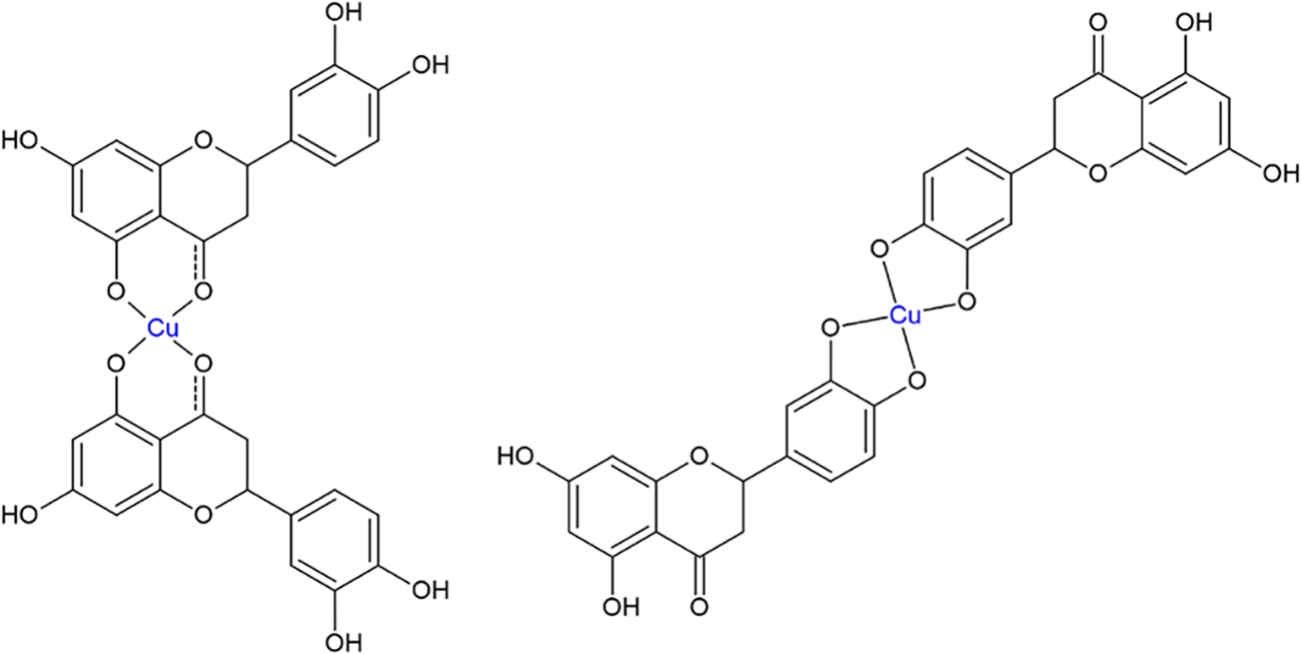
Possible structure representation of Cu^2+^- Flavonoid complexation (1:2).

#### 3.1.3 Scanning Electron Microscopy (SEM)

The SEM micrographs for propolis, CuCl_2_, and Cu-PRO are shown in Figure 7. According to Figure 7A, the propolis exhibits an amorphous morphology with the absence of ordered structures and porosity on the surface and irregularly shaped particles of varying sizes with a smooth surface. For CuCl_2_, Figure 7B shows large rectangular crystals with a smooth surface and a uniform distribution of sizes and shapes.

**Figure 7:**
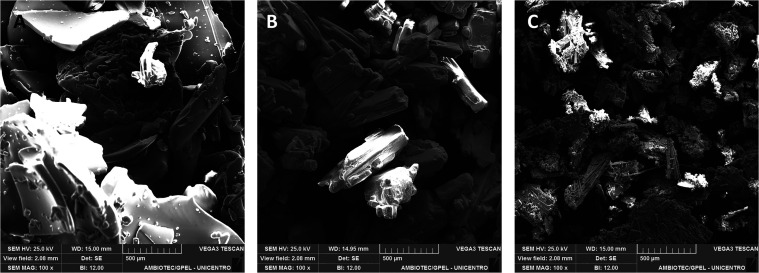
SEM micrographs at 100× magnification of (A) propolis, (B) CuCl_2_, and (C) Cu-PRO.

In Figure 7C, the morphology of the complex is observed, where clusters of small particles of different sizes have needle-like or rectangular crystalline structures. Additionally, particles with lower contrast are observed, characteristic of the organic part of propolis.

### 3.2 Antioxidant Activity

The antioxidant activity of the materials was evaluated using two metal ion reduction methodologies, CUPRAC (Cupric Reducing Antioxidant Capacity) and FRAP (Ferric Reducing Antioxidant Power), as other methods, such as radical scavenging like DPPH and ABTS, showed significant interference due to the presence of Cu^2+^. Figure 8 presents the obtained results.

**Figure 8:**
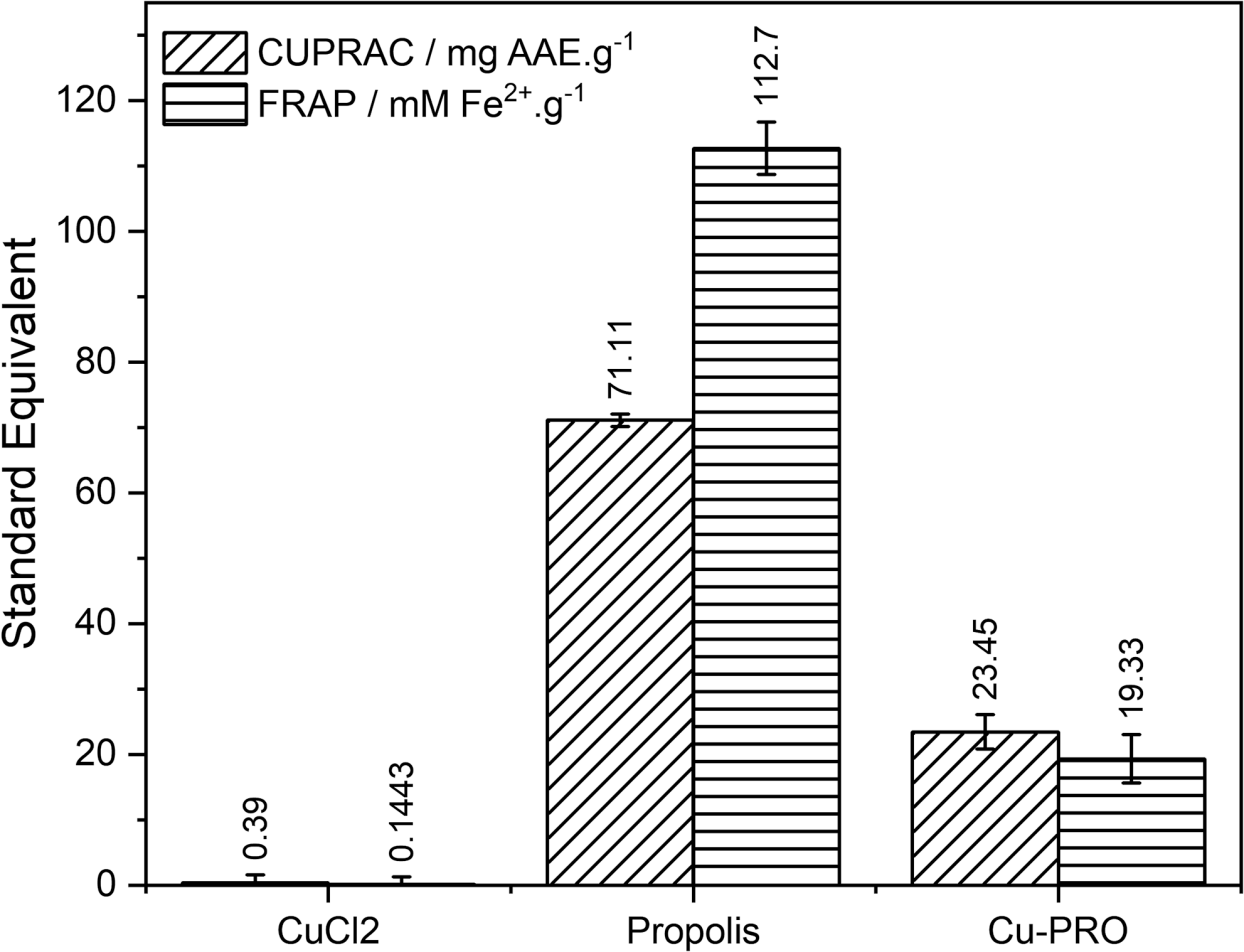
Antioxidant activities of the precursor materials and Cu-PRO in the FRAP and CUPRAC assay.

As expected, Cu^2+^ alone did not show significant antioxidant activity nor did it interfere with the measures, showing values below the LOD of the methods. On the other hand, the brown propolis extract exhibited high antioxidant activity, characteristic of its composition rich in phenolic compounds and flavonoids. However, the Cu-PRO complex showed significantly lower activity compared to pure propolis. This may be related to the complexation/interaction with the Cu^2+^ ion, which partially inhibits functional groups, leading to structural changes in the resonance network and reducing the antioxidant capacity of these compounds. Nevertheless, the complex retained considerable antioxidant activity, which could benefit therapeutic applications with a combined action of the effects of Cu^2+^ and propolis.

### 3.3 Antimicrobial Activity

Among the bacteria tested (Table 1), the propolis extract showed a strong antimicrobial activity against *E. coli* (12.5 μg·mL^–1^) and good activity against *L. monocytogenes* (50.0 μg·mL^–1^). At the same time, its action against *S. aureus* and *Salmonella gallinarum* was milder (100 μg·mL^–1^).

**Table 1:** MIC of Cu-PRO and Its Precursors against Pathogenic Bacterial Strains

	MIC (μg·mL^–1^)		
microorganism	propolis	CuCl_2_	Cu-PRO
*S. aureus*	100^a^	50.0^b^	50.0^a^
*L. monocytogenes*	50^b^	100^b^	100^b^
*S. gallinarum*	100^b^	50.0^b^	50.0^b^
*E. coli*	12.5^a^	100^b^	100^b^

a Legend: Bactericide effect (leads to cell death).

b Bacteriostatic effect (inhibit bacterial growth).

The antimicrobial activity of the tested propolis samples was particularly strong against *E. coli*, demonstrating remarkable potency compared to literature values, surpassing results from Gutiérrez-Gonçalves and Marcucci^40^ (MIC = 60.0 μg·mL^–1^) and Machado et al.^41^ (MIC = 31.2 μg·mL^–1^). This is notable given the inherent resistance of Gram-negative bacteria to propolis due to their complex outer membrane, suggesting potential chemical synergies or unique bioactive components in the tested sample. In complement, activity was against *S. aureus* contrasts with lower values found by Pamplona-Zomenhan et al.^42^ in ATTC strains (MIC_50_ = 1.420 μg·mL^–1^) for Brazilian propolis from Paraná State but were lower than some samples of Machado et al.^41^ (MIC = 156 μg·mL^–1^) of southern Brazilian propolis, possibly reflecting geographical variations in propolis composition.

For *L. monocytogenes*, the observed MIC is significantly lower than the mean values reported by Rendueles et al.^43^ (MIC = 97.66 μg·mL^–1^) for Spanish propolis under pH-modulated conditions. This analysis highlights potential geographical and methodological influences such as pH-dependent efficacy or variations in propolis chemistry in its bioactivity.

For CuCl_2_ and Cu-PRO, similar results were observed for *S. aureus* and *S. gallinarum*, with good inhibitory activity (50.0 μg·mL^–1^), but milder action against *E. coli* and *L. monocytogenes* (100 μg·mL^–1^). We highlight the activity against *S. aureus*, where Cu-PRO exhibited bactericidal action at a lower concentration than pure propolis extract, while CuCl_2_ showed bacteriostatic action. The complexation product was able to combine synergistically both activities of Cu^2+^ and propolis, for these bacteria, enhancing the effects of propolis against *S. aureus*. However, activity against *E. coli* and *L. monocytogenes* does not show any significant enhancement, when compared to the propolis extract, which exhibits a better inhibition capacity.

Copper compounds are widely recognized for their antimicrobial action, which is mediated by multiple synergistic toxicity mechanisms against microorganisms.^22,44^ These multifaceted mechanisms grant efficacy of copper as an antimicrobial agent in agricultural, industrial, and medical applications, although its use requires caution due to potential toxic and environmental impacts.^45,46^ According to Salah et al.,^22^ one of the primary mechanisms is the induction of oxidative stress through the generation of ROS by reduction of copper via a Fenton-like reaction. These ROS cause oxidative damage to critical cellular components, including membranes, proteins, and genetic material. Additionally, copper promotes genotoxicity by damaging microbial DNA.^44,47^ Finally, copper binding to functional protein groups (such as sulfhydryl, imidazole, and carboxyl groups) inactivates essential enzymes, disrupting key biochemical processes.^48^

### 3.4 Toxicological Modeling in *D. melanogaster*

Although copper is an essential micronutrient in metabolic processes, as discussed previously, it exhibits significant toxicity,^49^ at elevated concentrations by promoting the generation of ROS, increasing oxidative stress and leading cellular damage and cuproptosis mechanisms. Toxicological assessment is therefore fundamental to define the safe range between efficiency, adequate use, and toxicity, ensuring the development of safe applications.

*D. melanogaster* is a model organism widely used in initial toxicological screenings, including studies with copper compounds.^25,45,48–50^ Although its results cannot be directly extrapolated to mammals, this model allows for the robust identification of dose–response relationships and the detection of potential toxic and sublethal effects, providing essential preliminary data to guide, design, and justify subsequent studies and applications.

Survival analysis using the Kaplan–Meier Estimator and the log-rank test (Figure 9 and Table 2) indicates a significant overall difference in survival between Cu-PRO doses, indicating a clear dose-dependent relationship. Pairwise comparisons indicate that at the lower doses (0.1 and 1.3 mg·mL^–1^), no statistically significant differences were observed compared to the control (*p* > 0.05), indicating the absence of a significant toxic effect at these concentrations. However, starting at the 2.5 mg·mL^–1^ dose, for longer time exposure, a significant reduction in survival was observed compared to the control (*p* < 0.05, not shown in the table), suggesting the starting of the toxic effect.

**Figure 9:**
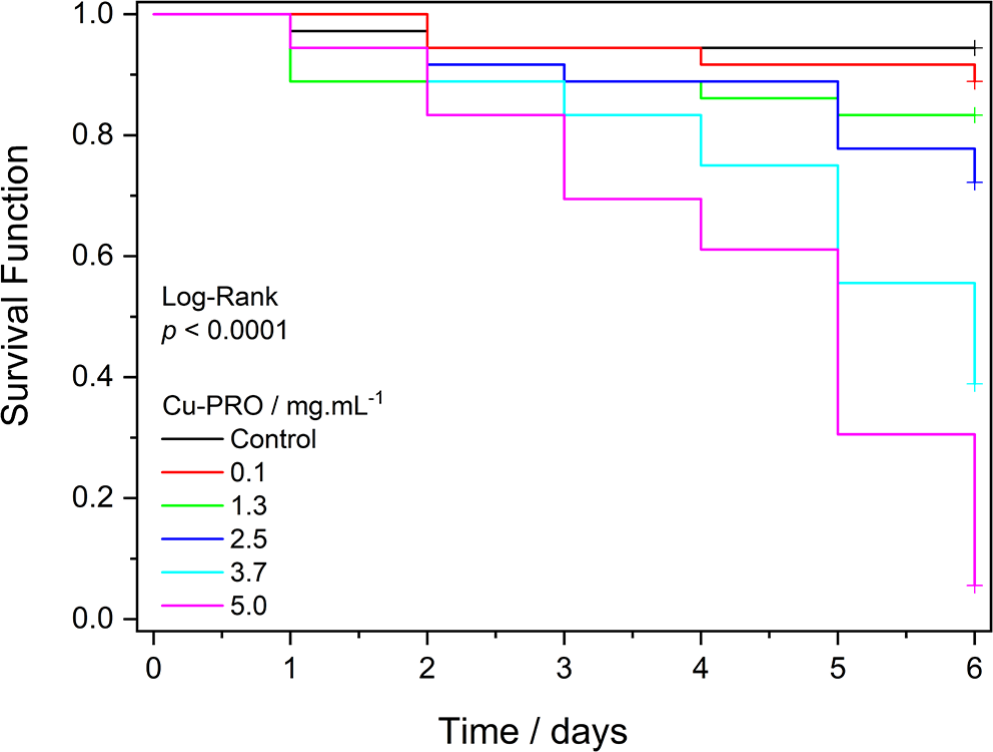
Kaplan–Meier survival curves by dose.

**Table 2:** Global and Pairwise Log-Rank Test Comparing Survival across Doses (In Pairwise Comparation, Only Non-Significant Differences, *p* > 0.05, Are Showed)

log-rank	χ^2^-value	*p*-value
global	100.2	<0.0001
0 vs 0.1	0.658	0.4172
0 vs 1.3	2.202	0.1378
0.1 vs 1.3	0.522	0.4700
0.1 vs 2.5	3.054	0.0805
1.3 vs 2.5	1.022	0.3120

This pattern is also clearly visualized in the Kaplan–Meier curves (Figure 9), in which the curves associated with the higher doses show steeper and earlier declines compared to the lower doses and the control, reflecting the progressive reduction in the probability of survival with increasing concentration.

Collectively, the results demonstrate a dose-dependent toxic effect, characterized by no significant mortality at lower doses (0.1–1.3 mg·mL^–1^), onset of toxicity at 2.5 mg·mL^–1^, and severe effects at higher doses. This pattern aligns with typical heavy metal toxicity mechanisms, where a threshold must be exceeded before oxidative stress or metal accumulation triggers irreversible damage.^24,48,51^

To quantify the effect of dose during the exposure time of Cu-PRO in the model organism, the adjustments of parametric survival models were evaluated. According to Table 3, the Generalized γ (GG) model was selected as the best-fit based primarily on the AIC, showing a substantial improvement (ΔAIC > 24) over all other parametric models tested. The variance-to-mean ratio of Cox-Snell residuals of the GG model was close to 1. All models showed a significant deviation from e^1^ distribution, failing in the Kolmogorov–Smirnov (KS) test, *p* < 0.05. This is an expected limitation when modeling discrete survival times, as it may affect the model’s predictive capacity, so all presented interpretation needs to be explicitly referred to the 6-day study period.

**Table 3:** Parametric Model AIC Comparison, and Cox-Snell Residual Analysis

model	AIC	log-lik	Cox-Snell Var/mean
Gen. γ	446.7	–218.40	0.9069
Weibull	470.7	–231.91	0.4588
standard γ	483.0	–238.50	0.3657
log–logistic	490.2	–241.68	0.3074
log-normal	508.7	–250.02	0.3540
exponential	507.1	–251.93	0.2377

Therefore, from all parametric models evaluated, the GG model was the one that best captured the complex nonmonotonic characteristic of the Cu-PRO induced hazard.

The GG model revealed statistically significant (*p* < 0.05) parameters (Table 4) that comprehensively describe the relationship between dose and survival time. The intercept parameter (β_0_ = 3.0768) indicates a baseline mean survival in logarithmic scale of time, in normal scale (*e* ^β_0_^) of approximately 21.7 days for the control group, serving as the baseline for modeling survival. The scale parameter (σ = 0.0732) demonstrates low variability in survival times, suggesting that the population shows a distribution and tolerance to Cu-PRO that tends to be close to the mean. The shape parameter (κ = 7.5727) indicates that the tail shape of the GG distribution differs from both log-normal (limit case κ → 0) and Weibull (κ = 1). Despite the wide CI_95%_ for κ, the *p*-value <0.05 already indicates a significant difference from κ = 0. This explains the better fit when compared to the AIC of Log-normal and Weibull models, even though the GG model presents greater complexity.

**Table 4:** Estimated Coefficients for the GG Survival Model^a^

coef	value	SE	CI_95%_	*p*-value
β_0_	3.077	0.198	2.6885; 3.4652	<0.001
β_1_	–0.2567	0.039	–0.3330; −0.1803	<0.001
σ	0.0732	0.035	0.0288; 0.1857	<0.001
κ	7.573	3.411	0.8869; 14.2586	0.026

a Legend: SE - Standard Error; CI95% - 95% Confidence interval.

Finally, the coefficient β_1_ associated with the effect of dose on the logarithm of time (β_1_ = −0.2567). Its negative value demonstrates that the Cu-PRO dose has an inverse relationship with survival time. This parameter determines the time ratio (*t*_R_ = *e* ^β_1_^), which indicates that for each increase in 1 mg·mL^–1^ dose (*x*), the survival time is reduced to approximately 77.36% (*t*_R_ = 0.7736) of the original time, equivalent to a 22.64% decrease in median lifespan per dose unit, demonstrating a clear quantitative dose-dependence of the complex toxic effect.

3 $$S\left(t\right)=1-\Gamma \left(\frac{1}{\kappa }{\left(\frac{\ln\left(t\right)-\left(\beta_{0}-\beta_{1}x\right)}{\sigma }\right)}^{\kappa }\right)$$

The simplified equation of the survival function in the GG model is described as eq 3,^52^ where Γ is the cumulative distribution function of the GG distribution. From the coefficients of the fitted model, the equation was used to determine the median lethal concentration (LC_50_) where the survival probability at a given time is *S*(*t*) = 0.5, and the Γ = 0.5.

According to Figure 10, it is observed that LC_50_ decreases progressively from 7.92 mg·mL^–1^ at *t* = 2 (CI_95%_ = 5.56; 10.3, wide range is related to lower mortality in this period) to 3.64 mg·mL^–1^ at *t* = 6 (CI_95%_ = 2.56; 4.72). This ∼54% reduction in the LC_50_ in 5 days highlights a cumulative effect of the compound in the studied period, where prolonged exposure significantly enhances toxicity.

**Figure 10:**
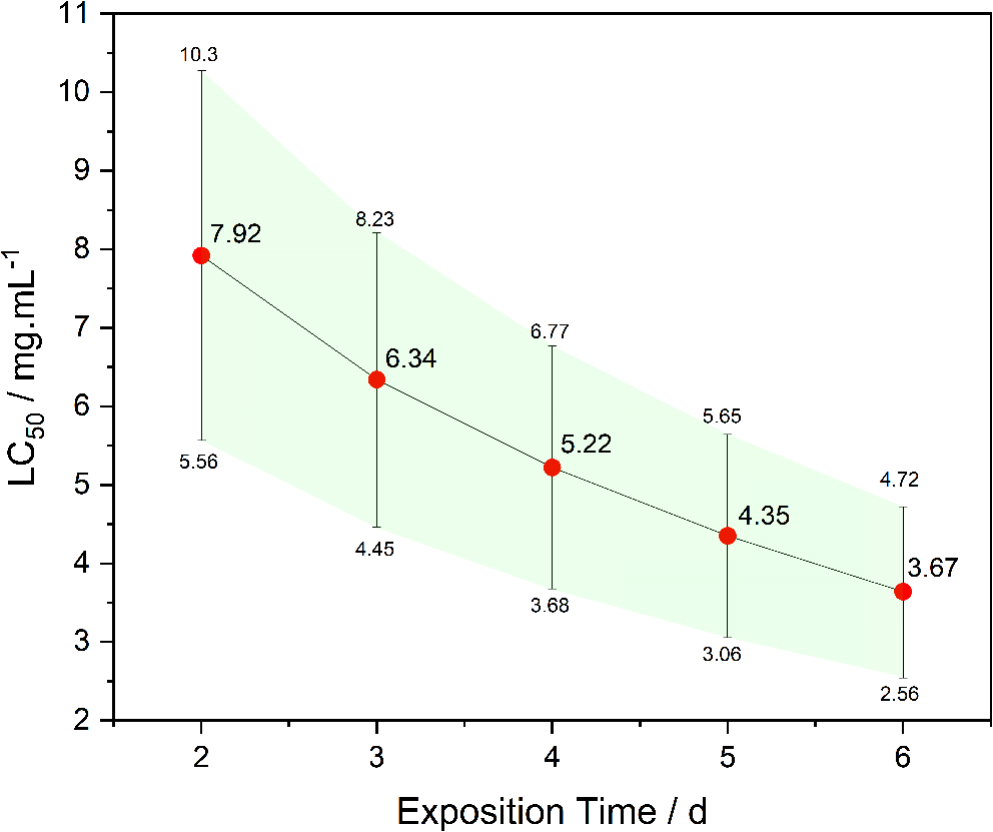
Chronic evolution of Cu-PRO LC_50_ over time.

In the literature, the toxicity of copper compounds has been widely studied in *D. melanogaster*.^25,45,47–51^ Although formal LC_50_ values are not common. Studies such as Klimaczewski et al.^53^ serve as a reference value involving toxicity of Cu^2+^. The author reports an LC_50_ of 3 mM Cu^2+^, in the form of CuSO_4_, (∼0.191 mg·mL^–1^ Cu^2+^) determined through a 4-day acute exposure, a reference cited and corroborated by Halmenschelager et al.^48^ and Ioan et al.^45^ Other studies have investigated copper toxicity over longer periods of time or in different chemical forms. Abolaji et al.^25^ evaluated both chronic (7 days at 1 mM Cu^2+^) and acute (24 h at 10 mM) exposures of Cu^2+^ chelated with D-penicillamine. The chelation led to prolonged survival compared to pure copper salt without mentioning quantitative values or comparative toxicity parameters. Meanwhile, Carmona et al.^44^ explored the effects of CuO-NPs (Copper oxide nanoparticles) and CuSO_4_ on larvae development, observing oxidative and genotoxic damage at concentrations ranging from 0.24 to 7.5 mg·mL^–1^.

Thus, using Klimaczewski et al.^53^ as a reference for LC_50_ of Cu^2+^, although, in a theoretically stoichiometric yield approximation, Cu-PRO tends to contains a relatively high amount of copper per gram (∼310 mgCu·g^–1^), its toxicity in *D. melanogaster* was significantly lower than that of reference free Cu^2+^. This reflects differences in the element’s bioavailability and the influence of chemical form on the metal’s toxic action. However, the true copper content in the final complex is a theoretical approximation, so this comparison may be limited in a qualitative way.

The significantly lower toxicity observed for the Cu-PRO complex compared to reference may be fundamentally linked to the intrinsic antioxidant properties of Brazilian brown propolis. Propolis is renowned for its rich composition of antioxidant compounds, particularly regarding a large diversity of flavonoids^4,26^ and phenolic acids (e.g., caffeoylquinic acids, *p*-cumaric acid),^41^ which exhibit a potent free radical scavenging capacity. Results shown in the antioxidant assays (Figure 8) demonstrate that the Cu-PRO complex retained significant antioxidant capacity despite the copper coordination. This suggests that the antioxidant components of propolis may effectively mitigate copper-induced oxidative stress within the *D. melanogaster*, delaying the toxicity.

The propolis ligands can not only coordinate Cu^2+^ but may also stabilize it in less reactive forms, reducing its capacity to participate in Fenton-like reactions, modulating generation of ROS. Also, the antioxidants in propolis could directly neutralize ROS produced during copper accumulation, protecting cellular components (lipids, proteins, DNA) from immediate oxidative damage. This might delay the onset of mortality and reduce acute toxicity, as observed in Kaplan–Meier survival curves (Figure 9) where significant effects only emerged at higher doses >2.5 mg·mL^–1^ in longer exposures.

This protective effect aligns with studies on other metal-chelating antioxidants (as described before in Abolaji et al.,^25^ studies) but highlights an advantage of propolis, its multicomponent composition offers a dual synergy, chelation plus antioxidant activity, potentially making it possibly more effective than synthetic chelators alone.

Consequently, the reduced bioavailability of free Cu^2+^ ions, combined with continuous ROS scavenging, likely explains the higher LC_50_ values of Cu-PRO compared to reference, and its delayed chronic toxicity profile, as seen in the time-dependent decrease in LC_50_.

## 4 Conclusions

The complexation of propolis with Cu^2+^ resulted in a new material with distinct physicochemical properties. UV–vis analysis showed enhanced absorption in the 800–900 nm region, suggesting potential for light-induced antibacterial applications. FTIR spectra indicated structural changes such as suppression of the carbonyl band, revealing possible copper interaction sites. Although Cu-PRO exhibited reduced antioxidant activity due to structural alterations from copper binding, it retained relevant activity for therapeutic potential. Its antimicrobial effect was partially enhanced against some of the bacteria tested, outperforming both pure propolis and CuCl_2_ against *S. aureus* and *S. gallinarum*, but no significant enhancement was observed in *E. coli* and *L. monocytogenes*.

Toxicological assessment in *D. melanogaster* revealed a dose-dependent chronic toxicity, with significant effects only at doses ≥2.5 mg·mL^–1^ in longer exposure scenarios. Parametric modeling showed nonmonotonic hazard with each dose unity reduced median longevity of the model organism by ∼23%, and LC_50_ decreased over time (from 7.92 mg·mL^–1^ at 2 days to 3.64 mg·mL^–1^ at 6 days), indicating cumulative toxicity. However, parametric modeling can only be evaluated within the 6-day study time, as the KS test, for all parametric models, does not follow a exact exponential distribution (*p* < 0.05). Despite that, Cu-PRO was highly less toxic than reference copper salt (LC_50_ = 3 mM; ∼0.191 mg·mL^–1^ of free Cu^2+^ in 4 days), likely due to chelation reducing metal bioavailability and propolis antioxidants mitigating oxidative stress.

These findings position Cu-PRO as a promising antimicrobial agent with an efficacy-safety balance. Future studies will explore visible-light-induced antimicrobial mechanisms, photodynamic effects, and synergism between photoactivation and controlled copper release to enhance its biological applications. This approach may pave the way for innovative applications of propolis as an antimicrobial and chelating agent, enhancing biological activities of metal complexes.

## References

[1] Anjum S. I., Ullah A., Khan K. A., Attaullah M., Khan H., Ali H., Bashir M. A., Tahir M., Ansari M. J., Ghramh H. A., Adgaba N., Dash C. K. (2019). Composition
and Functional Properties of Propolis (Bee Glue): A Review. Saudi J. Biol. Sci..

[2] Machado B., Pulcino T., Silva A., Melo D., Silva R., Mendonca I. (2016). Propolis as an Alternative
in Prevention and Control
of Dental Cavity. J. Apither..

[3] Pasupuleti V. R., Sammugam L., Ramesh N., Gan S. H. (2017). Honey, Propolis,
and Royal Jelly: A Comprehensive Review of Their Biological Actions
and Health Benefits. Oxid. Med. Cell. Longevity.

[4] Toreti V. C., Sato H. H., Pastore G. M., Park Y. K. (2013). Recent Progress
of Propolis for Its Biological and Chemical Compositions and Its Botanical
Origin. Evidence-Based Complementary Altern.
Med..

[5] Lopes G. A., Fidelis P. C., Almeida B. M. D., Almeida J. J., Ientz G. D. A. S., Binda N. S., Teixeira A. F., Vieira-Filho S. A., Caligiorne R. B., Saúde-Guimarães D. A., Brumano M. H. N., Figueiredo S. M. D. (2022). Antioxidant Activity, Sensory Analysis
and Acceptability of Red Fruit Juice Supplemented with Brazilian Green
Propolis. Food Sci. Technol..

[6] Zheng Y.-Z., Deng G., Liang Q., Chen D.-F., Guo R., Lai R.-C. (2017). Antioxidant Activity
of Quercetin and Its Glucosides
from Propolis: A Theoretical Study. Sci. Rep..

[7] Ahn J.-C., Biswas R., Chung P.-S. (2013). Synergistic
Effect of Radachlorin
Mediated Photodynamic Therapy on Propolis Induced Apoptosis in AMC-HN-4
Cell Lines via Caspase Dependent Pathway. Photodiagn.
Photodyn. Ther..

[8] Carvalho A. A., Finger D., Machado C. S., Schmidt E. M., Costa P. M. D., Alves A. P. N. N., Morais T. M. F., Queiroz M. G. R. D., Quináia S. P., Rosa M. R. D., Santos J. M. T. D., Pessoa C., Moraes M. O. D., Costa-Lotufo L. V., Sawaya A. C. H. F., Eberlin M. N., Torres Y. R. (2011). In Vivo Antitumoural
Activity and Composition of an Oil Extract of Brazilian Propolis. Food Chem..

[9] Kasote D., Bankova V., Viljoen A. M. (2022). Propolis:
Chemical Diversity and
Challenges in Quality Control. Phytochem. Rev..

[10] Doğan H., Silici S., Ozcimen A. A. (2020). Biological Effects of Propolis on
Cancer. Turk. J. Agric. Food Sci. Technol..

[11] Ibrahim N. I., Wong S. K., Mohamed I. N., Mohamed N., Chin K.-Y., Ima-Nirwana S., Shuid A. N. (2018). Wound Healing Properties of Selected
Natural Products. Int. J. Environ. Res. Public
Health.

[12] Bhargava P., Grover A., Nigam N., Kaul A., Doi M., Ishida Y., Kakuta H., Kaul S., Terao K., Wadhwa R. (2018). Anticancer Activity
of the Supercritical Extract of
Brazilian Green Propolis and Its Active Component, artepillin C: Bioinformatics
and Experimental Analyses of Its Mechanisms of Action. Int. J. Oncol..

[13] Olczyk P., Komosinska-Vassev K., Wisowski G., Mencner L., Stojko J., Kozma E. M. (2014). Propolis
Modulates Fibronectin Expression in the Matrix
of Thermal Injury. BioMed Res. Int..

[14] Henshaw F. R., Bolton T., Nube V., Hood A., Veldhoen D., Pfrunder L., McKew G. L., Macleod C., McLennan S. V., Twigg S. M. (2014). Topical Application
of the Bee Hive Protectant Propolis
Is Well Tolerated and Improves Human Diabetic Foot Ulcer Healing in
a Prospective Feasibility Study. J. Diabetes
Its Complications.

[15] Silva A. P. R., Soares A. P. S., Pessanha C. D. S., Roza C. M., Tavares L. S. C., Silva D. D. M., Cardoso M. M., Palermo T. A., Dos Santos C. M., Silva A. T. M. F. (2017). Uso Terapêutico
Da Pomada de Própolis
Em Diferentes Feridas Crônicas. Biol.
Saúde.

[16] Brandão M. G.
S. A., Ximenes M. A. M., Cruz G. S., Brito E. H. S., Veras V. S., Barros L. M., Araújo T. M. (2020). Terapia
Fotodinâmica No Tratamento de Feridas Infectadas Nos Pés
de Pessoas Com Diabetes Mellitus: Síntese de Boas Evidências:
Photodynamic Therapy in the Treatment of Infected Wounds on the Feet
of People with Diabetes Mellitus. Rev. Enferm.
Atual In Derme.

[17] Jin X., Xu H., Deng J., Dan H., Ji P., Chen Q., Zeng X. (2019). Photodynamic Therapy
for Oral Potentially Malignant Disorders. Photodiagn.
Photodyn. Ther..

[18] Hamblin M. R. (2016). Antimicrobial
Photodynamic Inactivation: A Bright New Technique to Kill Resistant
Microbes. Curr. Opin. Microbiol..

[19] Ribeiro I. S., Muniz I. P. R., Galantini M. P. L., Gonçalves C. V., Lima P. H. B., Silva N. R., De Oliveira S. L., Nunes M. S., Novaes A. K. S., De
Oliveira M. E. S., Costa D. J., Amaral J. G., Da Silva R. A. A. (2024). Antimicrobial
Photodynamic Therapy with Brazilian Green Propolis Controls Intradermal
Infection Induced by Methicillin-Resistant *Staphylococcus
aureus* and Modulates the Inflammatory Response in
a Murine Model. Photochem. Photobiol. Sci..

[20] Wang C.-C., Wang Y.-X., Yu N.-Q., Hu D., Wang X.-Y., Chen X.-G., Liao Y.-W., Yao J., Wang H., He L., Wu L. (2017). Brazilian Green Propolis Extract Synergizes with Protoporphyrin
IX-Mediated Photodynamic Therapy via Enhancement of Intracellular
Accumulation of Protoporphyrin IX and Attenuation of NF-κB and
COX-2. Molecules.

[21] Xu Y., Liu S., Zeng L., Ma H., Zhang Y., Yang H., Liu Y., Fang S., Zhao J., Xu Y., Ashby C. R., He Y., Dai Z., Pan Y. (2022). An Enzyme-Engineered
Nonporous Copper­(I)
Coordination Polymer Nanoplatform for Cuproptosis-Based Synergistic
Cancer Therapy. Adv. Mater..

[22] Salah I., Parkin I. P., Allan E. (2021). Copper as
an Antimicrobial Agent:
Recent Advances. RSC Adv..

[23] Guo Z., Chen D., Yao L., Sun Y., Li D., Le J., Dian Y., Zeng F., Chen X., Deng G. (2025). The Molecular
Mechanism and Therapeutic Landscape of Copper and Cuproptosis in Cancer. Signal Transduction Targeted Ther..

[24] Ji P., Wang P., Chen H., Xu Y., Ge J., Tian Z., Yan Z. (2023). Potential of Copper
and Copper Compounds
for Anticancer Applications. Pharmaceuticals.

[25] Abolaji A. O., Fasae K. D., Iwezor C. E., Farombi E. O. (2020). D-Penicillamine
Prolongs Survival and Lessens Copper-Induced Toxicity in *Drosophila melanogaster*. Toxicol.
Res..

[26] Finger D., Machado C. S., Torres Y. R., Quináia S. P., Thomaz A. C. G., Gobbo A. R., Monteiro M. C., Ferreira A. G., Sawaya A. C. H. F., Eberlin M. N. (2013). Antifungal Bioassay-Guided Fractionation
of an Oil Extract of Propolis. J. Food Qual..

[27] Mira L., Tereza Fernandez M., Santos M., Rocha R., Helena Florêncio M., Jennings K. R. (2002). Interactions of Flavonoids with Iron and Copper Ions:
A Mechanism for Their Antioxidant Activity. Free Radical Res..

[28] Kocot J., Kiełczykowska M., Luchowska-Kocot D., Kurzepa J., Musik I. (2018). Antioxidant
Potential of Propolis, Bee Pollen, and Royal Jelly: Possible Medical
Application. Oxid. Med. Cell. Longevity.

[29] Contieri L. S., De Souza Mesquita L. M., Sanches V. L., Chaves J., Pizani R. S., Da Silva L. C., Viganó J., Ventura S. P. M., Rostagno M. A. (2022). Recent
Progress on the Recovery of Bioactive Compounds Obtained from Propolis
as a Natural Resource: Processes, and Applications. Sep. Purif. Technol..

[30] Apak R., Güçlü K., Özyürek M., Karademir S. E. (2004). Novel Total Antioxidant Capacity Index for Dietary
Polyphenols and Vitamins C and E, Using Their Cupric Ion Reducing
Capability in the Presence of Neocuproine: CUPRAC Method. J. Agric. Food Chem..

[31] Rufino, M. S. M. ; Alves, R. E. ; Brito, E. S. ; Morais, S. M. ; Sampaio, C. G. ; Pérez-Jiménez, J. ; Saura-Calixto, F. D. Determinação Da Atividade Antioxidante Total Em Frutas Pelo Método de Redução Do Ferro (FRAP), Comunicado Técnico Embrapa, 2006; pp 1–4.

[32] NCCLS . Methods for Dilution Antimicrobial Susceptibility Tests for Bacteria That Grow Aerobically: Approved Standard, 6th ed.; NCCLS: Wayne, PA, 2003.

[33] Reference Method for Broth Dilution Antifungal Susceptibility Testing of Yeats: Approved Standard, 3rd ed.; Clinical and Laboratory Standards Institute: Wayne, PA, 2008.

[34] Kumari P., Ain U., Firdaus H. (2023). A Reliable and Consistent Method to Quantify Percent
Lethality and Life Span in *Drosophila melanogaster*. Bio-Protoc..

[35] Paganotti R., Rezende J., Barbeira P. (2014). Discrimination Between Producing
Regions of Brazilian Propolis by UV–VIS Spectroscopy and Partial
Least Squares Discriminant Analysis. Curr. Anal.
Chem..

[36] Tomazzoli M. M., Pai Neto R. D., Moresco R., Westphal L., Zeggio A. R. S., Specht L., Costa C., Rocha M., Maraschin M. (2015). Discrimination
of Brazilian Propolis According to the Seasoning Using Chemometrics
and Machine Learning Based on UV-Vis Scanning Data. J. Integr. Bioinform..

[37] Barbeira P. J. S., Paganotti R. S. N., Ássimos A. A. (2013). Development
of a Multivariate Calibration Model for the Determination of Dry Extract
Content in Brazilian Commercial Bee Propolis Extracts through UV–Vis
Spectroscopy. Spectrochim. Acta, Part A.

[38] Scatolini, A. M. Estudo de hidroxiapatita contendo própolis de origem brasileira: caracterização, atividade antimicrobiana e efeito citotóxico dos materiais, Dissertartação de Mestrado em Engenharia e Ciência dos Materiais; Universidade de São Paulo (USP): Pirassununga, SP, 2017. teses.usp.br/teses/disponiveis/74/74133/tde-20092017-094523/ (accessed March 21, 2025).

[39] Redondo, G. D. P. Desenvolvimento, caracterização e estudos de dissolução de microencapsulados poliméricos de liberação controlada de extrato de própolis vermelha obtidos através da técnica de secagem Spray Drying, Dissertartação de Mestrado em Ciências Farmacêuticas; Universidade Federal de Alagoas: Maceió, AL, 2018. repositorio.ufal.br/handle/riufal/6705 (accessed March 21, 2025).

[40] Marcucci M. C., Marcucci M. C. (2009). Atividades Antimicrobiana e Antioxidante da Própolis
do Estado do Ceará. Rev. Fitos.

[41] Machado C. S., Finger D., Dias De
Lima T. C., Modesto T., Monteiro M. C., De Oliveira F. D. C. E., Pessoa C., Torres Y. R. (2021). Seeking the Regional
Identity of Brown Propolis Produced in Southern Brazil and Linked
to Total Levels of Bioactive Compounds and Biological Activity. ACS Food Sci. Technol..

[42] Pamplona-Zomenhan L.
C., Pamplona B. C., Silva C. B. D., Marcucci M. C., Mimica L. M. J. (2011). Evaluation
of the in Vitro Antimicrobial Activity of an Ethanol Extract of Brazilian
Classified Propolis on Strains of *Staphylococcus aureus*. Braz. J. Microbiol..

[43] Rendueles E., Mauriz E., Sanz-Gómez J., Adanero-Jorge F., García-Fernandez C. (2023). Antimicrobial Activity
of Spanish
Propolis against *Listeria monocytogenes* and Other Listeria Strains. Microorganisms.

[44] Carmona E. R., Inostroza-Blancheteau C., Obando V., Rubio L., Marcos R. (2015). Genotoxicity of Copper
Oxide Nanoparticles in *Drosophila melanogaster*. Mutat.
Res., Genet. Toxicol. Environ. Mutagen..

[45] Ioan S., Irina P., Emilian O., Sorina P., Cerasela P., Adriana C., Dorin C., Alina-Maria T.-C., Dacian L., Ciprian S., Anamaria
Aurelia M., Laura-Gratiela V., Mariana G. (2024). Application of the *Drosophila melanogaster* Research Model to Evaluate
the Toxicity Levels between Lead and Copper. Appl. Sci..

[46] Tortella G., Rubilar O., Fincheira P., Parada J., De Oliveira H. C., Benavides-Mendoza A., Leiva S., Fernandez-Baldo M., Seabra A. B. (2024). Copper Nanoparticles as a Potential Emerging Pollutant:
Divergent Effects in the Agriculture, Risk-Benefit Balance and Integrated
Strategies for Its Use. Emerging Contam..

[47] Everman E. R., Cloud-Richardson K. M., Macdonald S. J. (2021). Characterizing the Genetic Basis
of Copper Toxicity in *Drosophila* Reveals a Complex
Pattern of Allelic, Regulatory, and Behavioral Variation. Genetics.

[48] Halmenschelager P. T., Da Rocha J. B. T. (2019). Biochemical CuSO4 Toxicity in *Drosophila
melanogaster* Depends on Sex and Developmental Stage
of Exposure. Biol. Trace Elem. Res..

[49] Zamberlan D. C., Halmenschelager P. T., Silva L. F. O., Da Rocha J. B. T. (2020). Copper Decreases
Associative Learning and Memory in *Drosophila melanogaster*. Sci. Total Environ..

[50] Baeg E., Sooklert K., Sereemaspun A. (2018). Copper Oxide
Nanoparticles Cause
a Dose-Dependent Toxicity via Inducing Reactive Oxygen Species in
Drosophila. Nanomaterials.

[51] Hanumanthappa R., Venugopal D. M., P C N., Shaikh A., B M S., Heggannavar G. B., Patil A. A., Nanjaiah H., Suresh D., Kariduraganavar M. Y., Raghu S. V., Devaraju K. S. (2023). Polyvinylpyrrolidone-Capped
Copper Oxide Nanoparticles-Anchored Pramipexole Attenuates the Rotenone-Induced
Phenotypes in a *Drosophila* Parkinson’s Disease
Model. ACS Omega.

[52] Lawless, J. F. Statistical Models and Methods for Lifetime Data, 1st ed.; Wiley Series in Probability and Statistics; Wiley, 2002.

[53] Klimaczewski C. V., Ecker A., Piccoli B., Aschner M., Barbosa N. V., Rocha J. B. T. (2018). Peumus Boldus Attenuates Copper-Induced Toxicity in *Drosophila melanogaster*. Biomed.
Pharmacother..

